# Results of a project to calibrate mercury sphygmomanometer blood pressure-measuring devices in Egypt

**DOI:** 10.1038/s41371-020-00424-0

**Published:** 2020-10-09

**Authors:** Soliman Ghareeb, Ghada Youssef, Haytham Soliman Ghareeb, Hazem Abd El-Mageed, Muhammad H. Mesalm, Remon Talaat, Alaaeldin Eltawil, Doaa M. Hasan, M. Mohsen Ibrahim

**Affiliations:** 1 Cardiology Department, Cairo University, Cairo, Egypt; 2 Cardiology Department, El Fayoum University, El Fayoum, Egypt; 3 Ipsos Healthcare, Cairo, Egypt; 4 National Institute of Standards (NIS), Cairo, Egypt

**Keywords:** Diagnosis, Hypertension

## Abstract

Hypertension (HTN) is a common health problem and a major cardiovascular risk factor. Accurate measurement of blood pressure (BP) is mandatory for proper diagnosis and follow-up. The aim of this study was to evaluate the accuracy of mercury sphygmomanometer BP-measuring devices in public hospitals in Cairo, Egypt. Fifty public hospitals were included, and 10% of all mercury sphygmomanometer devices in each hospital were tested. Assessment included physical condition (e.g., mercury status, lid of the device, state of the rubber tubes), leakage rate, and calibration accuracy (as compared with a reference device). Devices were approved as accurate when they could successfully pass all three assessment tests. The total number of sphygmomanometer devices was 465. The overall pass rate was 1.3% (six devices). Twenty-five (5.2%) devices passed all of the physical tests, 50 (10.8%) passed the leakage test, and 50 (16.5%) passed the calibration accuracy test. There were 162 (34.8%) devices that showed a high leakage rate (>80 mmHg) and thus were not tested for calibration accuracy. In conclusion, most of the mercury sphygmomanometer devices in hospitals are neglected and not checked regularly for any errors. A plan should be made to gradually replace those failed devices with new, validated, and well-calibrated devices, preferably devices that do not contain mercury.

## Introduction

According to the World Health Organization (WHO), one in every eight deaths is caused by hypertension (HTN), making HTN one of the leading killers worldwide. Globally, one billion people are hypertensive, and four million people die annually as a direct result of HTN. From 2005 to 2015, the death rate attributable to HTN increased by 10.5%, and the actual number of deaths attributable to high blood pressure (BP) rose by 37.5% [1].

HTN in Egypt is one of the most common noncommunicable diseases [2]. Based on data from the Egyptian National Hypertension Project (NHP; 1992–1995), the national estimation of the prevalence of HTN in Egypt is 26.3% [3]. Since then, no other nationwide projects have been executed. The most recent WHO report on noncommunicable diseases in Egypt indicated that the prevalence of HTN rose to 40% [4].

Evidence for the diagnosis and management of high BP is based on studies following the recommendations for accurate measurement of BP. The practice of evidence-based medicine requires accurate measurement of BP in clinical practice. The mercury sphygmomanometer was once considered the most popular device and the standard of care for indirect BP measurement. Nowadays, many countries have stopped using this device because of the fear of mercury-related environmental health hazards. However, in Egypt, mercury sphygmomanometer devices are still the most widely used devices for measuring BP.

Precise estimation of BP requires the use of an accurate sphygmomanometer, which should be checked and calibrated regularly [5]. It is recommended that sphygmomanometers used to measure BP be maintained and calibrated regularly [6–9], to guarantee that the pressure scale remains accurate to within the standard of ±3 mmHg [10]. Unfortunately, the accuracy of these devices is an often overlooked aspect of the quality of the diagnostic process of HTN.

In the era of busy clinics, regular calibration of sphygmomanometers is usually overlooked or neglected. The purpose of this study is to evaluate the condition and validity of mercury sphygmomanometers commonly used in some public hospitals located in Cairo governate, Egypt.

## Methods

This is a cross-sectional, multicenter, analytical study involving hospitals located in Cairo governorate. Fifty hospitals were randomly selected as a representative sample (level of 95% and confidence interval/error range of ±10%). In each hospital, we evaluated 10% of the available sphygmomanometer devices.

We assessed the devices on site in the chosen hospitals. Calibration followed the recommendations of ISO 81060-1:2007 [11] and the OIML R 16-1: 2002 [12].

The reference device was an electronic pressure gauge (DPG-102, Dwyer, Michigan City, IN, USA) that had an accuracy of ±0.25 mmHg (range, 0–775 mmHg) [13]. To ensure that pressure accuracy was maintained, the reference device/pressure gauge was calibrated at the National Institute of Standards laboratory, Cairo, Egypt, on commencement and completion of the study.

The evaluation of each device included the following three steps physical assessment, air leakage rate of the pneumatic system, and cuff pressure indication (calibration accuracy). For a device to be considered as approved or verified, it must have successfully passed all the applicable tests.

### Physical assessment

The physical assessment of the mercury devices is explained in Table 1.

**Table. 1:** Physical assessment tests for mercury sphygmomanometer BP-measuring devices.

*Status item*	*Device was considered defective if any of the following was present*
The Mercury	—Black discoloration or air bubbles of the mercury. —Mercury was not sufficient to be at the zero mark on the tube (when zero pressure applied with an open system and a vertical sphygmomanometer.
The lid of the device	—Not secure in its position. —Not effectively closed.
Indicators and displays (Scale and glass tube clarity)	—Not clear or not easy to be read. —Glass tube discoloration or contamination.
The leather washer (The leather disc) or filters	—Mercury loss test is positive. After stopping pumping, mercury continues to rise and does not stop. The dropping speed time from 200 mmHg to 0 mmHg is more than 1 s. —The rapid exhaust test from 200 mmHg to 40 mmHg is more than 1.5 s
Zero Reading	—Deviated reading by >±2 mmHg.
Health and safety	—Absence of the cuff correct positioning indicator over the artery. —Absence of the cuff limb circumference indicator for which it is appropriate. —Absence of instructions regarding its use, maintenance, and safety.
The cuffs	—Rips, tears or inability to be fastened and to stay fastened on inflation.
The bladder	—Worn, torn or prolapsed out of the cuff.
The inflation bulb	—Cracks, leaking air on pumping.
The rubber tubing	—Holes, leaks, cracks, or excessive wear.
Connectors	—Loose connections.

### Leakage test

During this assessment, the reference device measured the loss of pressure by the sphygmomanometer device over 1 min. After checking that all connections were tightly fitted and that the bulb was securely closed, the leak rate function of the reference device was selected, and the cuff was manually inflated to 260 mmHg. A 1-min countdown timer was used by the observer to record the starting pressure (0 s) and the resulting cuff pressure after 1 min, as well as the difference between the two. To pass the test, the difference had to be less than or equal 4 mmHg per minute.

There was no need for reference standard used (RSU) for the leak test, because the column of the unit under calibration (UUC) was used to determine the leak. The leak test was performed at 260 mmHg, using a pressure hand pump (PHP) to generate the required pressure. The shut off valve was then used to check the rate of change of the UUC readings over time (1 min). Devices with a pressure leak greater than 80 mmHg were considered faulty units and were not eligible for the next step of the evaluation, the calibration accuracy assessment.

### Calibration accuracy assessment

Calibration took place by directly comparing the UUC (i.e., mercury sphygmomanometer) readings with those of the RSU. The RSU is a digital pressure indicator with full scale of 775 mmHg, resolution of 0.1 mmHg, and accuracy of 0.25%. A PHP was used to generate the testing pressure points. The PHP includes a volume adjuster that enables fine pressure control and a bleed valve [13].

During this assessment, the RSU, UUC, and PHP were directly connected on a rigid, stable, straight surface to carry out the different pressure tests. The RSU was calibrated, traceable to National Institute of Standards primary standard [14]. The UUC was installed as near to the RSU as possible, taking into consideration adjustment for the head difference between the two of these.

To avoid bias, the digital display of the RSU was covered so as to blind the observer to the reference pressures. For a device to be approved or verified, the maximum permissible error for measurement of the cuff pressure at any point of the scale range had to be ±0.4 kPa (3 mmHg) for mercury sphygmomanometers.

### Protocol of hospital contact

Hospitals were contacted to obtain permission through formal letters of invitation of collaboration, delivered personally to fully elaborate any unclear point. A total of 61 hospitals were approached, of which 50 agreed to participate (rejection rate of 18%), and the calibration service was provided on site.

### Sample size

This study included 465 devices from 50 hospitals representing eight sectors of hospitals in greater Cairo, Egypt. The eight sectors included all types of medical practice in greater Cairo, excluding private and military hospitals. Sample size calculation was based on a total universe of 95 hospitals in Cairo (nonprivate nor military) and with consideration of a confidence level of 95% and confidence interval/error range of ±9.59%. A sample size of 50 hospitals was included. Hospitals were randomly selected from each sector and included both small and large hospitals.

Mercury sphygmomanometer devices were randomly selected from different departments in each hospital (inpatient, outpatient, emergency, etc.). The sample of selected devices represented 10% of all hospital devices. In small hospitals, where the entire number of devices was <10, all devices were included in the calibration analysis process. An inquiry for a previous calibration of the selected devices was obtained from the records of the technical support departments of the institutions.

### Data entry

The design of the data entry process was built on Excel (Microsoft) to show different study variables; double data entry was performed to ensure no data entry mistakes were done. Random data checks were done before proceeding to data analysis. Data were then exported to SPSS for analysis. Reliability analysis was performed on the data to ensure its consistency, and the reliability index was determined to be high.

### Statistical analysis

Data were statistically described in terms of mean and standard deviation, range, or frequencies (number of cases) and percentages when appropriate. All statistical calculations were performed using computer program SPSS (Statistical Package for the Social Science; SPSS Inc., Chicago, IL, USA) version 19 for Microsoft Windows.

## Results

A total of 465 mercury sphygmomanometer BP-measuring devices were randomly selected and evaluated. Only 13 (2.8%) devices had undergone maintenance before, but there were no records on the kind of maintenance performed. The records mentioned only that the devices had been tested before, with no specific technical details provided. Only six (1.3%) of the devices had successfully passed the combined three assessment parameters.

### Physical assessment of device parts

**Fig. 1:**
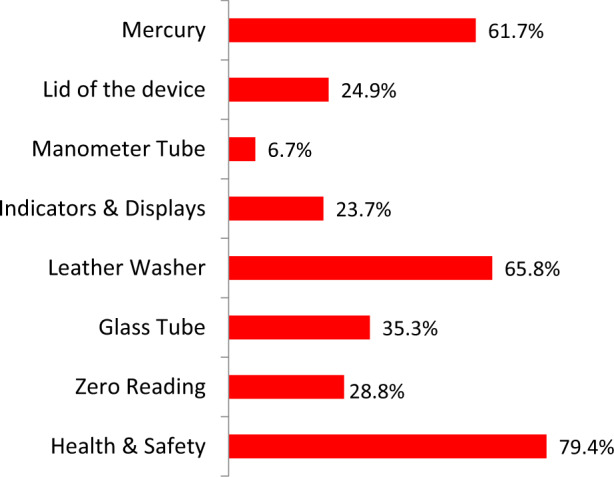
Failure rate at the different check points in the physical assessment of the devices.

**Fig. 2:**
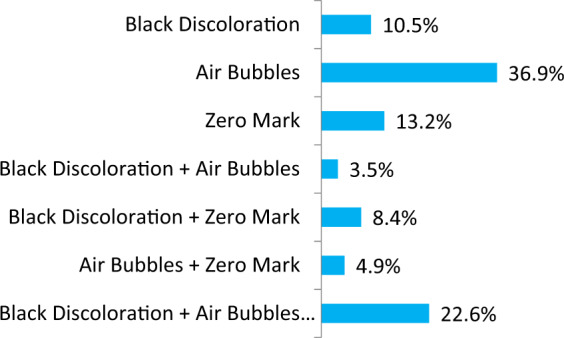
Frequency of the different reasons of failed mercury check in the tested devices.

**Fig. 3:**
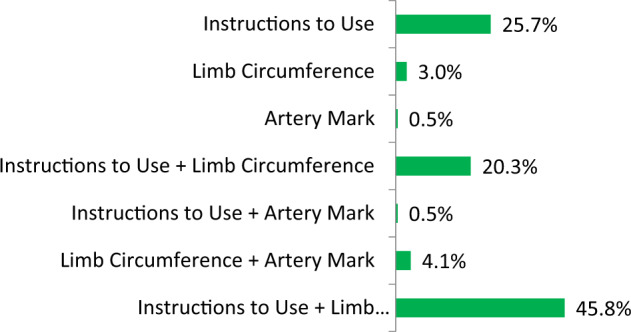
Frequency of the different reasons of failed health and safety check in the tested devices.

Of the 465 sphygmomanometers devices, only 25 (5.2%) passed all points of physical assessment. Figs. 1–3 present the failure rate for each item of the physical assessment.

### Leakage rate

On performing the leakage test, we determined that 162 (34.8%) devices had very high levels of leakage (>80 mmHg). Because it was impractical to assess the calibration accuracy in devices with such a high leakage rate (too high to permit static pressure readings), these devices were not tested for leakage, and they were included in the failure group. Most of the other tested devices (*n* = 303) failed the leakage test (*n* = 253, 83.5%).

### Calibration accuracy assessment

Only devices that were eligible for leakage assessment were tested for calibration accuracy (*n* = 303, 65.2%). After testing the devices at all five different pressure points (50, 100, 150, 200, 250 mmHg) up and down scales, only 78 (16.5%) devices met the standard of ±3 mmHg. The smallest pressure point to be successfully met was at 250 mmHg down scale (only 68.3% of devices). Generally, the devices tended to down estimate all of the pressure points.

## Discussion

The diagnosis of HTN depends on repeated office BP measurements using standard, validated sphygmomanometer devices [15]. Although the European Union has recommended phasing out mercury sphygmomanometer devices, these devices are still widely accepted in Egypt as the gold standard for BP measurement. Not only in Egypt, but in many other developing countries, mercury devices are still the standard of care for BP measurement [16, 17]. The reason for the delay in replacing hazardous mercury devices with nonmercury ones (auscultatory and oscillometric) in developing countries is related to the cost of the new devices and the impact of this cost on the limited resources of health care systems. Nonmercury devices also require calibration at regular intervals, thus adding an extra cost to the process of BP measurement.

Accurate BP measurement is critical for the proper diagnosis and follow-up of hypertensive patients. Accurate measurement requires well-trained personnel, standardized measuring techniques, and validated and properly calibrated devices [18].

In Egypt, regular examination and calibration of BP-measuring devices is not a common trend. The aim of this project was to raise concerns about the importance of the regular evaluation of mercury sphygmomanometer BP-measuring devices through testing routinely used devices in some of the public hospitals in Cairo, Egypt.

Fifty public hospitals representing different health care systems were chosen and invited to participate in the project. From each hospital, 10% of the available sphygmomanometer devices were randomly selected and tested for physical well-being as well as for leakage and calibration accuracy. A total of 465 devices were tested, and only six (1.3%) devices successfully passed the three assessment tests. This result is not different from what Knight et al. [19] had found: none of their 472 tested sphygmomanometer devices complied fully with the British Sphygmomanometer Standard current in 2001. A more recent study showed that one in seven devices failed the standards of the British Hypertension Society (within <3 mmHg of the true value) [20]. Many other studies showed different levels of inaccurate mercury sphygmomanometer devices ranging from 1 to 28% [19, 21–24].

In Australia, the recommended frequency for physical evaluation of mercury sphygmomanometers is every 6 months, whereas calibration is recommended every 36 months for sphygmomanometers that are fixed to immovable objects and every 12 months for portable sphygmomanometers [25]. In this study, only 2.8% of the tested devices had been checked previously, and the records of the timing and the results of the check were not available. This may reflect either ignorance of the importance of maintaining these devices or reluctance to do so.

The increase in the direct costs caused by calibration of sphygmomanometers was found to be negated by the cost reduction that would have been obtained from the reduction in additional office visits of every patient [26]. Assessment of the mercury devices required two trained technicians, who can complete a device checkup in just 10–15 min, using a reference device that costs about 7000–8000 LE. The relatively inexpensive reference device and the short duration of the assessment as well as the feasibility of training medical personnel make the assessment of the devices a practical solution to the present situation.

However, the problem is not only when and how to maintain the devices; the problem is what to do when devices fail the assessment tests. The decision to throw away all of these failed devices and replace them with new ones is very difficult and impractical. Yet, the consequences of erroneous BP measurement can affect the health care system in an even more serious way. It was shown that a consistent 5-mmHg error in diastolic pressure can more than double or halve the number of patients diagnosed with diastolic HTN, whereas a consistent 5-mmHg error in systolic pressure may result in underdiagnosis of systolic HTN by 30% or overdiagnosis by 43% [25, 27].

For these reasons, sincere efforts should be made to gradually replace these failed devices in hospitals with new devices that do not contain mercury. The high percentage of devices with a leakage carries high environmental risks, because mercury is a highly toxic substance. Physicians in private clinics, pharmacies, and individuals who have home BP-measuring devices should all be aware of the importance of device maintenance. Those individuals do not need to buy the reference device for calibration; rather, they can check their devices at a certified calibration center located in different Egyptian governorates.

### Limitations of the study

We did not include private hospitals and clinics, pharmacies, and outpatient clinics, which serve a large sector of patients, in this study because of some logistic issues. In addition, the project did not include hospitals from other regions in Egypt, where the situation could possibly be worse.

## Conclusion

There is a lack of regular assessment of BP-measuring devices in Egypt. Failure to maintain these devices can negatively affect the health care system as well as individual patients. Erroneous measurement may lead to either overestimation of BP values, resulting in unnecessary treatments given to healthy individuals with associated higher financial burden on the health care system, or underestimation of BP values, which leads to missing truly hypertensive patients and sending them home without proper treatment, exposing them to the entire spectrum of HTN complications. This project revealed a serious problem that requires immediate and well-planned campaigns to raise awareness and find solutions for the current situation.

### Summary table

#### What is known about this topic

- Mercury BP-measuring devices are still used on a large scale in some countries, including Egypt.
- Mercury BP-measuring devices should be calibrated at regular intervals.
- Failure to calibrate these devices my lead to errors in BP measurement.

#### What this study adds

- Most of the mercury BP-measuring devices in Egyptian public hospitals are not meeting the standards of good devices.
- Most public hospitals were either unaware of the importance of calibrating the devices or negligent to do so.
- Governmental efforts should be made to replace the mercury-containing BP-measuring devices by non-mercury ones.
- Mercury devices which are still in action need regular and accurate calibration.
